# Adaptive Problem Solving Dynamics in Gate-Model Quantum Computers

**DOI:** 10.3390/e24091196

**Published:** 2022-08-26

**Authors:** Laszlo Gyongyosi

**Affiliations:** 1 Department of Networked Systems and Services, Budapest University of Technology and Economics, 1117 Budapest, Hungary; gyongyosi@hit.bme.hu; 2 MTA-BME Information Systems Research Group, Hungarian Academy of Sciences, 1051 Budapest, Hungary

**Keywords:** gate-model quantum computer, quantum computer, quantum computing

## Abstract

Gate-model quantum computer architectures represent an implementable model used to realize quantum computations. The mathematical description of the dynamical attributes of adaptive problem solving and iterative objective function evaluation in a gate-model quantum computer is currently a challenge. Here, a mathematical model of adaptive problem solving dynamics in a gate-model quantum computer is defined. We characterize a canonical equation of adaptive objective function evaluation of computational problems. We study the stability of adaptive problem solving in gate-model quantum computers.

## 1. Introduction

As the development of quantum computers evolves significantly [1,2,3,4,5,6,7,8,9,10,11,12,13,14,15,16,17,18,19,20,21,22,23,24,25,26,27], a fundamental need to characterize the attributes of problem solving in quantum computers has arisen. Gate-model quantum computers have particular relevance since most of these architectures allow for practical solutions to be implemented on near-term settings. Another fundamental application of gate-model quantum computers lies in the quantum devices of the quantum Internet [28]. In a gate-model quantum computer, the computational steps are realized via unitary gates. The gates are associated with a gate parameter value, while the computational problem fed into the quantum computer identifies an objective function [6,7,8,9,10,29] (objective function examples can be found in [7,9,10,11,14,15]). The aim of problem solving is to maximize the objective function value via several iteration steps. Each iteration step includes the application of unitary gates as well as a measurement of the resulting quantum states. From the measurement results, an averaged value can be determined to estimate the actual objective function value [7]. The problem solving method, therefore, is identified via a series of well-defined computational steps. However, the dynamical attributes of a procedure for adaptive problem solving and objective function evaluation in a gate-model quantum computer environment are still not well defined.

Many hybrid quantum variational circuits also use gate-model circuits and classical objective functions [30,31,32,33]. Particularly, our system model utilizes the quantum approximate optimization algorithm (QAOA) [6,7], which is a variational quantum algorithm, such as [31,34,35]. Variational quantum algorithms are useful for machine learning problems [11,36] or combinatorial optimization [37,38]. The problem resolution dynamics of these quantum variational circuits also identifies an adaptive model; therefore, a perspective of adaptive models will be useful for investigating and optimizing variational quantum algorithms in near-term implementations. In a general approach, the aim of a variational quantum algorithm is to produce an entangled output quantum state via a quantum computer, the state of which represents the answer to an input computational problem. An input quantum state fed into the quantum computer evolves via the quantum circuit of the quantum computer where the quantum gates are unitary operators with a particular gate parameter value (control parameters). In the quantum computer, these gate parameter values change dynamically, and these values adapt to the particular objective function value of the input problem in each iteration step (therefore, “varied” dynamically and adaptively).

Here, we define a mathematical model of adaptive problem solving dynamics in gate-model quantum computers. Adaptive dynamics (AD) [39,40,41,42,43] is a quantitative approach to analyzing evolutionary processes with several application areas. The aim of the proposed model is to characterize the dynamical attributes of adaptive problem solving via iterative objective function maximization, by utilizing the framework of adaptive dynamics [39]. The model defines the stability of the objective function evolution procedure in each iteration step. The objective function stability evaluates the convergence of an objective function component associated with a given unitary to a target value. For a stable objective function component, the iteration converges to an optimal (maximized) value that results in the maximization of an objective function of the computational problem fed into the quantum computer. We show that, in the computational stage (i.e., before a measurement), the stability function of the objective function is in a superposition of stable and unstable states. We reveal the dynamical attributes of the stability of the objective function components. We define a canonical equation of adaptive problem solving dynamics in a gate-model quantum computer. The canonical equation describes the evolution of the objective function components in the dynamical model as a selection gradient toward an optimal solution.

From a physical and engineering perspective, the proposed results are interpreted as follows. In an experimental gate-model quantum computer setting, the aim of the problem solving is to find the gate parameter values of the quantum computer’s unitaries. The evolution of the output quantum state of the quantum computer represents the answer to the problem if the objective function value associated with a particular input problem is high enough. Alternatively, the maximization of an objective function is analogous to the minimization of a Hamiltonian, and the output quantum state can be a ground state of a Hamiltonian model in near-term experimental settings. Our results reveal that the adaptive problem solving dynamics can be defined via the canonical equations of the quantum computer’s unitaries, since an objective function component is determined by the gate parameter of a given unitary. The adaptive procedure for generating the output quantum state of a gate-model quantum computer can be characterized by a set of classical control parameters that are varied dynamically and adaptively during the iteration steps. The proposed results are, therefore, directly applicable to near-term quantum computer implementations and gate-model quantum devices, allowing for a widespread application of our model in different physical and engineering problems.

The novel contributions of our manuscript are as follows:

1. A mathematical model of adaptive problem solving dynamics is defined for gate-model quantum computers. The proposed model characterizes the dynamical attributes of adaptive problem solving via iterative objective function maximization.
2. A canonical equation of adaptive problem solving dynamics is derived for objective function maximization in a gate-model quantum computer (variational quantum algorithm).
3. We define the stability of the problem solving steps to reach a maximized target value of the objective function. The stability of the objective function evaluation is associated with the gate errors in the hardware level of the gate-model quantum computer.

This paper is organized as follows. Section 2 proposes the problem statement and the system model. Section 3 defines the stability function of the objective function evaluation. Section 4 derives the canonical equation of adaptive objective function evaluation. Section 5 studies the superposition of the stability functions. Finally, Section 6 concludes the results. Supplemental information is included in Appendix A.

## 2. Problem Statement and System Model

### 2.1. Problem Statement

The problems to be solved are summarized in Problems 1–3.

**Problem** **1.** *Let R be the number of measurement rounds needed to evaluate the averaged objective function $\tilde{C}=\frac{1}{R}\sum_{r=1}^{R}C\left(z^{r}\right),$ where r is the measurement round, r=1,…,R; $C\left(z^{r}\right)$ is the objective function associated with an r-th measurement round; and $z^{r}$ is an n-length bit string. In a particular r-th measurement round, an $|s\rangle$ input quantum state is prepared and fed into the quantum computer, and the $|\phi \rangle$ output state of the quantum computer is measured via a measurement M to determine $C\left(z^{r}\right)$. Define the stability of the objective function evaluation in a measurement round r. Show that, if the objective function is stable for an objective function component, then the objective function component converges to an optimum in the r-th iteration step.*

**Problem** **2.** *Let $C_{i}^{r-1}=C^{r-1}\left(U_{i}\right)$ be an objective function component associated with an i-th unitary $U_{i}$ in an $\left(r-1\right)$-th measurement round, defined as $C_{i}^{r-1}=\frac{1}{2}\left(1-z_{i}^{r-1}\right)$, where $z_{i}^{r-1}$ refers to an i-th bit of $z^{r-1}$, $z_{i}^{r-1}\in \left\{-1,1\right\}$. Then, let $f_{\varepsilon }\left(\cdot \right)$ be a function, and $f_{\varepsilon }\left(C^{r-1}\left(U_{i}\right)\right)$ be a canonical equation that describes the evolution of the $C^{r-1}\left(U_{i}\right)$ objective function component in the dynamical model as a selection gradient toward an optimal solution. Find the canonical equation of $f_{\varepsilon }\left(C^{r-1}\left(U_{i}\right)\right)$.*

**Problem** **3.** *Prove that, in a gate-model quantum computer, the stability functions formulate a superposition in the computational stage.*

The solutions to Problems 1–3 are proposed in Theorems 1–3.

### 2.2. System Model

The sequence of *L* unitaries [7] of the quantum computer is defined as

(1) $$U\left(\vec{\theta }\right)=U_{L}\left(\theta_{L}\right)U_{L-1}\left(\theta_{L-1}\right)\ldots U_{1}\left(\theta_{1}\right),$$

where $\vec{\theta }$ is the *L*-dimensional vector of the gate parameters of the unitaries (gate parameter vector),

(2) $$\vec{\theta }={\left(\theta_{1},\ldots ,\theta_{L}\right)}^{T},$$

and an *i*-th unitary gate $U_{i}\left(\theta_{i}\right)$ is evaluated as

(3) $$U_{i}\left(\theta_{i}\right)=\exp\left(-i\theta_{i}P_{i}\right),$$

where $P_{i}$ is a generalized Pauli operator acting on a few quantum states (qubits in an experimental setting) formulated by the tensor product of Pauli operators $\left\{\sigma_{x},\sigma_{y},\sigma_{z}\right\}$[7]. Note that $U(\vec{\theta })$ in (1) identifies a unitary resulting from the serial application of the *L* unitary operators $U_{L}\left(\theta_{L}\right)U_{L-1}\left(\theta_{L-1}\right)\ldots U_{1}\left(\theta_{1}\right)$ and for an input quantum state $|\varphi \rangle$:

(4) $$U\left(\vec{\theta }\right)|\varphi \rangle =U_{L}\left(\theta_{L}\right)U_{L-1}\left(\theta_{L-1}\right)\ldots U_{1}\left(\theta_{1}\right)|\varphi \rangle .$$

In a qubit setting, the gate structure of the quantum computer integrates *a* single-qubit and *b* two-qubit unitaries, L=a+b, where a *j*-th single-qubit gate implements an $X_{j}=\sigma_{x}^{j}$ operator, while a two-qubit gate between qubits *j* and *k* realizes a $Z_{j}Z_{k}=\sigma_{z}^{j}\sigma_{z}^{k}$ operator [7].

Let *C* be a particular objective function of an optimization problem subject of a maximization via the quantum computer. Then, the $U(\vec{\theta })$ sequence from (1) can be evaluated as

(5) $$U\left(\vec{\theta }\right)=U\left(B,\vec{\beta }\right)U\left(C,\vec{\gamma }\right),$$

where

(6) $$U\left(B,\vec{\beta }\right)=\prod_{j}U\left(B_{j},\beta_{j}\right)=\prod_{j=1}^{a}U\left(B_{j},\beta_{j}\right),$$

where $\vec{\beta }$ is the gate parameter vector of the *a* single-qubit unitaries,

(7) $$\vec{\beta }={\left(\beta_{1},\ldots ,\beta_{a}\right)}^{T},$$

while *B* is defined as a sum

(8) $$B=\sum_{j=1}^{a}X_{j}=\sum_{j=1}^{a}\sigma_{x}^{j},$$

where $X_{j}$ refers the Pauli *X* operator, $\sigma_{x}$, in a *j*-th 1-qubit gate. Thus, $U(B_{j},\beta_{j})$ from (6) can be defined as

(9) $$U\left(B_{j},\beta_{j}\right)=\exp\left(-i\beta_{j}X_{j}\right),$$

where $B_{j}=X_{j}$, while the two-qubit unitaries are defined as

(10) $$U(C,\vec{\gamma })=\prod_{\langle jk\rangle }U(C_{jk},\gamma_{jk})=\prod_{\langle jk\rangle =1}^{b}U(C_{jk},\gamma_{jk}),$$

where $\langle jk\rangle$ is a physical connection between qubits *j* and *k* in the hardware-level of the quantum computer, $\gamma_{jk}$ is the gate parameter of the two-qubit gate $Z_{j}Z_{k}=\sigma_{z}^{j}\sigma_{z}^{k}$ between qubits *j* and *k*. Then, the $\vec{\gamma }$ gate parameter vector of the *b* two-qubit unitaries is as

(11) $$\vec{\gamma }={\left(\gamma_{1},\ldots ,\gamma_{b}\right)}^{T},$$

where $C_{jk}$ is a component of the objective function and unitary $U\left(C_{jk},\gamma_{jk}\right)$ for a given $\langle jk\rangle$ is defined as

(12) $$U\left(C_{jk},\gamma_{jk}\right)=U\left(Z_{j}Z_{k},\gamma_{jk}C_{jk}\right)=\exp\left(-i\gamma_{jk}C_{jk}Z_{j}Z_{k}\right),$$

where

(13) $$Z_{j}Z_{k}=\sigma_{z}^{j}\sigma_{z}^{k}.$$

At a particular physical connectivity of the quantum computer, $C\left(z\right)$ is defined as the sum of all objective function components between all $\langle jk\rangle$, as

(14) $$C\left(z\right)=\sum_{\forall \langle jk\rangle }C_{jk}\left(z\right),$$

where $C_{jk}\left(z\right)$ is the objective function component evaluated for a given $\langle jk\rangle$ (objective function component $C_{jk}\left(z\right)$ is a part of the objective function $C\left(z\right)$, which is also part of the parametrized quantum circuits (PQC), such that $\langle jk\rangle$ identifies a physical connection between qubits *j* and *k* in the hardware of the gate-model quantum computer), while *z* is an *n*-length bitstring,

(15) $$z=z_{1}z_{2}\ldots z_{n},$$

where $z_{i}$ identifies an *i*-th bit, $z_{i}\in \left\{-1,1\right\}$.

For a given *z*, a *n* qubit length $|z\rangle$ computational basis state is defined as

(16) $$|z\rangle =|z_{1}z_{2}\ldots z_{n}\rangle ,$$

from which the $|s\rangle$ input state of the quantum computer is set as

(17) $$|s\rangle =\frac{1}{\sqrt{2^{n}}}\sum_{z}|z\rangle .$$

The *n* qubit length $|\phi \rangle$ output state of the quantum computer is as

(18) $$\begin{array}{cc} |\phi \rangle & =U\left(\vec{\theta }\right)|s\rangle \\ \; & =U\left(B,\vec{\beta }\right)U\left(C,\vec{\gamma }\right)|s\rangle . \end{array}$$

The output state (18) is measured via a measurement array *M* to determine $C\left(z\right)$ (14).

In the space–time volume of a gate-model quantum computer, we assume that an array of qubits is arranged on a grid of a particular size. In the gate structure, a layer of 1-qubit gates and a layer of 2-qubit gates act on the qubits (see also (5)). These 1-qubit and 2-qubit layers can be applied in *p* rounds, formulating a *p*-level quantum circuit.

For a level-*p* circuit, a set of *p* gate parameter vectors, $\vec{\beta }$ and $\vec{\gamma }$, are set, as

(19) $${\vec{\beta }}^{\left(1\right)},\ldots ,{\vec{\beta }}^{\left(p\right)},$$

and

(20) $${\vec{\gamma }}^{\left(1\right)},\ldots ,{\vec{\gamma }}^{\left(p\right)};$$

therefore, a *p*-level circuit *U* is defined as

(21) $$U=U\left(B^{\left(p\right)},{\vec{\beta }}^{\left(p\right)}\right)U\left(C^{\left(p\right)},{\vec{\gamma }}^{\left(p\right)}\right)\cdots U\left(B^{\left(1\right)},{\vec{\beta }}^{\left(1\right)}\right)U\left(C^{\left(1\right)},{\vec{\gamma }}^{\left(1\right)}\right).$$

For simplicity, in (18), we used p=1; however, the results can be extended for an arbitrary *p* [7].

For a *p*-level quantum circuit, the unitaries of the layers are set via gate parameter vectors (19) and (20), respectively. By using the state $|s\rangle$ (17) as input of the quantum computer, the 1-qubit gates in (5) are set to the Pauli *X* operators [7], since the input state $|s\rangle$ is an eigenstate of each *X* with eigenvalue 1.

**Figure 1:**
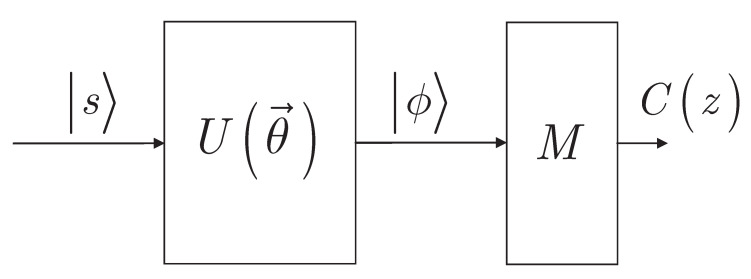
System model. An *n*-length input quantum state $|s\rangle$ (17) is fed into the $U(\vec{\theta })$ unitary structure (5) of the gate-model quantum computer. The $|\phi \rangle$ output quantum state (18) is measured via a measurement array *M*. The *M* measurement array represents a measurement in the computational basis to produce the *n*-length string *z* (15) from the *n* qubit length output state $|\phi \rangle$ (18) to evaluate the objective function value $C\left(z\right)$ (14).

The system model at p=1 is depicted in Figure 1.

### 2.3. Objective Function

The aim of running the quantum computer is to produce an output state $|\phi \rangle$ with a high value of some classical objective function *C*. The maximization of *C* is made via the selection of the gate parameters of the unitaries of the QG quantum gate structure of the gate-model quantum computer.

The *C* classical objective function can be interpreted as a sum of over individual terms, defined on *n*-bit bit strings $z=z_{1}z_{2}\cdots z_{n}$, as

(22) $$C\left(z\right)=\sum_{\alpha =1}^{m}C_{\alpha }\left(z\right),$$

where $C_{\alpha }\left(z\right)$ is clause; *m* is the number of clauses; and $C_{\alpha }\left(z\right)$ acts on a small subset of the bits, defined as

(23) $$C_{\alpha }\left(z\right)=\left\{\begin{array}{c} 1,\;\mathrm{if}\;z\;\mathrm{satisfies}\;\mathrm{clause}\;\alpha \\ 0,\;\mathrm{otherwise} \end{array}.\right.$$

Let $|s\rangle$ be the input state of the quantum computer from (17). Since $|s\rangle$ is an eigenstate of $X_{1}X_{2}\ldots X_{n}$ and operator *X* commutes with all of the unitaries in the system model, the $|\phi \rangle$ output state in (18) formulates an *n*-qubit entanglement [7], that can be rewritten as

(24) $$|\phi \rangle =|w\rangle +|\bar{w}\rangle ,$$

where *w* is an *n*-length bit string, while $\bar{w}$ is the inverse (bit-flip) of *w*.

Let *w* be an arbitrary *n*-length bit string. Then, for a particular *z*, the $C\left(z\right)$ objective function subject of a maximization can be defined as

(25) $$C\left(z\right)=-\mathrm{Ham}\left(z,w\right)\left(n-\mathrm{Ham}\left(z,w\right)\right)+{\left(\frac{n}{2}\right)}^{2},$$

where $\mathrm{Ham}\left(\cdot \right)$ is the Hamming distance [7], defined between binary strings *z* and *w*, as follows:

(26) $$\mathrm{Ham}\left(z,w\right)=f_{c}\left(z⊕w\right),$$

where ⊕ is the XOR operation, while $f_{c}\left(\cdot \right)$ is a function that returns the total number of ones in the resultant string $\left(z⊕w\right)$.

For a *p*-level circuit (for a *p*-level quantum circuit, the 1-qubit and 2-qubit gate layers are applied for *p* rounds), the $C\left(z\right)$ objective function in (25) can be maximized via the selection of the gate parameter vectors (19) and (20). If p=1, the maximization is made via gate parameter vectors (7) and (11), while for p>1, the gate parameter vectors are defined (19) and (20).

## 3. Stability of Objective Function Evaluation

In the system model, the stability of the objective function evaluation is associated with the gate errors in the hardware level of the gate-model quantum computer. If gate errors occur, then the actual quantum gates do not correspond perfectly to the desired gate parameter values, and the output state of the quantum computer becomes distorted. In our system model, the error of the gate parameter values also models the decoherence on the hardware level of the quantum computer, since the decoherence also leads to degradation of the actual output state of the quantum computer. Due to physical-level errors, the measured string values and the objective function values become distorted. In the mathematical model, the errors and noise are associated with the errors in the gate parameter values of the unitaries; therefore, the physical-level source of the actual error is irrelevant.

**Theorem** **1.** *(Stability of the objective function evaluation). The stability of the objective function components determines the convergence of the objective function to a target value. The stability depends on the gate parameters of the unitaries of the gate-model quantum computer.*

**Proof.**  Let $C\left(z^{r}\right)$ be the objective function evaluated via a string $z^{r}$ of an *r*-th measurement round, r=1,…,R, as

(27) $$C\left(z^{r}\right)=\sum_{\forall \langle ij\rangle }C_{\langle ij\rangle }^{r}\left(z^{r}\right),$$

where $C_{\langle ij\rangle }^{r}\left(z^{r}\right)$ is an objective function component associated with unitaries $U_{i}$ and $U_{j}$, as

(28) $$C_{\langle ij\rangle }^{r}\left(z^{r}\right)=\frac{1}{2}\left(1-z_{i}^{r}z_{j}^{r}\right),$$

where $z_{i}^{r}$, $z_{j}^{r}$ refer to an *i*-th and *j*-th bit of $z^{r}$, $z_{i}^{r}\in \left\{-1,1\right\}$, $z_{j}^{r}\in \left\{-1,1\right\}$; which is decomposable as

(29) $$C_{\langle ij\rangle }^{r}\left(z^{r}\right)=\left(\frac{1}{2}-\frac{1}{2}z_{i}^{r}\right)+z_{i}^{r}\frac{1}{2}\left(1-z_{j}^{r}\right)=C_{i}^{r}+z_{i}^{r}C_{j}^{r},$$

where $C_{i}^{r}$ is an objective function component associated with $U_{i}$ in an *r*-th measurement round, as

(30) $$C_{i}^{r}=\frac{1}{2}\left(1-z_{i}^{r}\right)=C^{r}\left(U_{i}\right)$$

and $C_{j}^{r}$ is an objective function component defined for $U_{j}$ in an *r*-th measurement round, as

(31) $$C_{j}^{r}=\frac{1}{2}\left(1-z_{j}^{r}\right)=C^{r}\left(U_{j}\right).$$

By utilizing the framework of adaptive dynamics [39], for a particular unitary sequence

(32) $$U\left({\vec{\theta }}^{r-1}\right)=U_{L}^{r-1}\left(\theta_{L}^{r-1}\right)U_{L-1}^{r-1}\left(\theta_{L-1}^{r-1}\right)\ldots U_{1}^{r-1}\left(\theta_{1}^{r-1}\right)$$

of *L* unitaries in an $\left(r-1\right)$-th measurement round, we define the $\alpha^{r-1}\left({\vec{\theta }}^{r-1}\right)$ objective function component vector, as

(33) $$\alpha^{r-1}\left({\vec{\theta }}^{r-1}\right)={\left(C^{r-1}\left(U_{1}\right),\ldots ,C^{r-1}\left(U_{L}\right)\right)}^{T},$$

while for

(34) $$U\left({\vec{\theta }}^{r}\right)=U_{L}^{r}\left(\theta_{L}^{r}\right)U_{L-1}^{r}\left(\theta_{L-1}^{r}\right)\ldots U_{1}^{r}\left(\theta_{1}^{r}\right),$$

the objective function components are set in the vector

(35) $$\beta^{r}\left({\vec{\theta }}^{r}\right)={\left(C^{r}\left(U_{1}\right),\ldots ,C^{r}\left(U_{L}\right)\right)}^{T}.$$

Let $N_{QG}^{r-1}$ be the vector of the total $n_{QG}$, $n_{QG}\geq L$ objective function components (equals the total unitary operators of the quantum computer) at an $\left(r-1\right)$-th measurement round,

(36) $$N_{QG}^{r-1}={\left(C^{r-1}\left(U_{1}\right),\ldots ,C^{r-1}\left(U_{n_{QG}}\right)\right)}^{T},$$

from which $\kappa^{r-1}$ is defined as

(37) $$\kappa^{r-1}=N_{QG}^{r-1}-\alpha^{r-1}\left({\vec{\theta }}^{r-1}\right),$$

while for an *r*-th measurement round, we set $\tau^{r}$ as

(38) $$\tau^{r}=N_{QG}^{r}-\beta^{r}\left({\vec{\theta }}^{r}\right),$$

for the remaining objective function components of QG.

Then, let $\theta_{i}^{r}\in \left[0,\pi \right]$ be the gate parameter of unitary $U_{i}$ in an *r*-th measurement round, and let $C^{r}\left(U_{i}\right)$ the objective function component associated with $U_{i}$ in an *r*-th measurement round, updated via function $f\left(C^{r}\left(U_{i}\right)\right)$ as

(39) $$f\left(C^{r}\left(U_{i}\right)\right)=p_{i}^{r}C^{r-1}\left(U_{i}\right)+\left(1-p_{i}^{r}\right)C^{r}\left(U_{i}\right),$$

where $C^{r-1}\left(U_{i}\right)$ is the objective function component if $U_{i}=I$, while $C^{r}\left(U_{i}\right)$ is an updated objective function component if $U_{i}\neq I$, and

(40) $$p_{i}^{r}=\cos^{2}\left(\theta_{i}^{r}\right)$$

and

(41) $$1-p_{i}^{r}=\sin^{2}\left(\theta_{i}^{r}\right).$$

Then, using (40) and (41), the formula of (39) can be rewritten as

(42) $$f\left(C^{r}\left(U_{i}\right)\right)=\cos^{2}\left(\theta_{i}^{r}\right)A+\sin^{2}\left(\theta_{i}^{r}\right)B,$$

where

(43) $$A=C^{r-1}\left(U_{i}\right)={\bar{X}}_{i}^{r-1}+\rho_{i}^{r-1}\cos\left(\theta_{i}^{r}\right)$$

and

(44) $$B=C^{r}\left(U_{i}\right)={\bar{X}}_{i}^{r}+\rho_{i}^{r}\sin\left(\theta_{i}^{r}\right),$$

where $\bar{X}$ and ρ are some constants.

To evaluate the objective function component stability, some basic terms are defined as follows.

Let $J\left(C^{r-1}\left(U_{i}\right),\tau^{r}\right)$ be a Jacobian matrix [39] at a particular $C^{r-1}\left(U_{i}\right)$ and $\tau^{r}$, defined as

(45) $$J\left(C^{r-1}\left(U_{i}\right),\tau^{r}\right)=\left(\begin{array}{cc} J_{a} & J_{b}\\ J_{c} & J_{d} \end{array}\right),$$

where

(46) $$J_{a}={\tilde{\alpha }}^{r-1}\left({\vec{\theta }}^{r-1}\right)\frac{\partial }{\partial \alpha^{r-1}\left({\vec{\theta }}^{r-1}\right)}f\left(\alpha^{r-1}\left({\vec{\theta }}^{r-1}\right),0,{\tilde{\kappa }}^{r-1},C^{r-1}\left(U_{i}\right),\cdot ,\tau^{r}\right)$$

(47) $$J_{b}={\tilde{\alpha }}^{r-1}\left({\vec{\theta }}^{r-1}\right)\frac{\partial }{\partial \kappa^{r-1}}f\left({\tilde{\alpha }}^{r-1}\left({\vec{\theta }}^{r-1}\right),0,\kappa^{r-1},C^{r-1}\left(U_{i}\right),\cdot ,\tau^{r}\right)$$

(48) $$J_{c}=\frac{\partial }{\partial \alpha^{r-1}\left({\vec{\theta }}^{r-1}\right)}F\left(\alpha^{r-1}\left({\vec{\theta }}^{r-1}\right),0,{\tilde{\kappa }}^{r-1},C^{r-1}\left(U_{i}\right),\cdot ,\tau^{r}\right)$$

and

(49) $$J_{d}=\frac{\partial }{\partial \kappa^{r-1}}F\left({\tilde{\alpha }}^{r-1}\left({\vec{\theta }}^{r-1}\right),0,\kappa^{r-1},C^{r-1}\left(U_{i}\right),\cdot ,\tau^{r}\right),$$

where ${\tilde{\alpha }}^{r-1}\left({\vec{\theta }}^{r-1}\right)$ and ${\tilde{\kappa }}^{r-1}$ refer to vectors $\alpha^{r-1}\left({\vec{\theta }}^{r-1}\right)$ and $\kappa^{r-1}$ at equilibrium states of the $\alpha^{r-1}{\left({\vec{\theta }}^{r-1}\right)}^{\prime }$ and ${\kappa^{r-1}}^{\prime }$ derivatives of $\alpha^{r-1}\left({\vec{\theta }}^{r-1}\right)$ and $\kappa^{r-1}$, such that relations

(50) $$\begin{array}{c} \alpha^{r-1}\left({\vec{\theta }}^{r-1}\right)={\tilde{\alpha }}^{r-1}\left({\vec{\theta }}^{r-1}\right)\\ \kappa^{r-1}={\tilde{\kappa }}^{r-1} \end{array}$$

hold for (45), while · refers to any objective function component value, while functions $f\left(\cdot \right)$ and $F\left(\cdot \right)$ evaluate the $\alpha^{r-1}{\left({\vec{\theta }}^{r-1}\right)}^{\prime }$ and ${\kappa^{r-1}}^{\prime }$ derivatives of $\alpha^{r-1}\left({\vec{\theta }}^{r-1}\right)$ and $\kappa^{r-1}$, as

(51) $$\begin{array}{cc} \; & f\left(\alpha^{r-1}\left({\vec{\theta }}^{r-1}\right),\beta^{r}\left({\vec{\theta }}^{r}\right),{\tilde{\kappa }}^{r-1},C^{r-1}\left(U_{i}\right),C^{r}\left(U_{i}\right),\tau^{r}\right)\\ \; & \equiv \frac{1}{L}\left(\lambda_{1}-\lambda_{2}\right), \end{array}$$

where

(52) $$\lambda_{1}=\left|C^{r-1}\left({\vec{\theta }}^{r-1}\right)>0\right|$$

identifies the number of non-zero $C^{r-1}\left(U_{1},\ldots ,U_{L}\right)$ objective function components in an $\left(r-1\right)$-th measurement round taken over unitaries $U_{1},\ldots ,U_{L}$, while

(53) $$\lambda_{2}=\left|C^{r-1}\left({\vec{\theta }}^{r-1}\right)=0\right|,$$

refers to the number of zero $C^{r-1}\left(U_{1},\ldots ,U_{L}\right)$ objective function components of an $\left(r-1\right)$-th measurement round taken over $U_{1},\ldots ,U_{L}$, while function

(54) $$F\left(\alpha^{r-1}\left({\vec{\theta }}^{r-1}\right),\beta^{r}\left({\vec{\theta }}^{r}\right),{\tilde{\kappa }}^{r-1},C^{r-1}\left(U_{i}\right),C^{r}\left(U_{i}\right),\tau^{r}\right)={\kappa^{r-1}}^{\prime }$$

returns the derivate ${\kappa^{r-1}}^{\prime }$.

Then, let S be the state space defined as

(55) $$S≐\left(\alpha^{r-1}\left({\vec{\theta }}^{r-1}\right),\beta^{r}\left({\vec{\theta }}^{r}\right),\kappa^{r-1}\right),$$

with an equilibrium state $\tilde{S}$, as

(56) $$\tilde{S}≐\left({\tilde{\alpha }}^{r-1}\left({\vec{\theta }}^{r-1}\right),0,{\tilde{\kappa }}^{r-1}\right),$$

which at $\alpha^{r-1}\left({\vec{\theta }}^{r-1}\right)=0$ results in

(57) $$\beta^{r}{\left({\vec{\theta }}^{r}\right)}^{\prime }=\beta^{r}\left({\vec{\theta }}^{r}\right)f\left(\beta^{r}\left({\vec{\theta }}^{r}\right),0,\kappa^{r-1},C^{r}\left(U_{i}\right),\cdot ,\tau^{r}\right)$$

and

(58) $${\kappa^{r-1}}^{\prime }=F\left(0,\beta^{r}\left({\vec{\theta }}^{r}\right),\kappa^{r-1},\cdot ,C^{r}\left(U_{i}\right),\tau^{r}\right).$$

Then, the $J_{\tilde{S}}\left(C^{r-1}\left(U_{i}\right),C^{r}\left(U_{i}\right),\tau^{r}\right)$ Jacobian matrix [39] at the equilibrium of (56) can be defined as

(59) $$J_{\tilde{S}}\left(C^{r-1}\left(U_{i}\right),C^{r}\left(U_{i}\right),\tau^{r}\right)=\left[\begin{array}{cc} J\left(C^{r-1}\left(U_{i}\right),\tau^{r}\right) & \ldots \\ 0 & f\left(0,{\tilde{\alpha }}^{r-1}\left({\vec{\theta }}^{r-1}\right),,{\tilde{\kappa }}^{r-1},C^{r}\left(U_{i}\right),C^{r-1}\left(U_{i}\right),\tau^{r}\right) \end{array}\right]$$

with an eigenvalue

(60) $$\lambda \left(C^{r-1}\left(U_{i}\right),C^{r}\left(U_{i}\right),\tau^{r}\right)=f\left(0,{\tilde{\alpha }}^{r-1}\left({\vec{\theta }}^{r-1}\right),{\tilde{\kappa }}^{r-1},C^{r}\left(U_{i}\right),C^{r-1}\left(U_{i}\right),\tau^{r}\right),$$

which identifies the fitness function, while $J\left(C^{r-1}\left(U_{i}\right),\tau^{r}\right)$ is given in (45).

Then, from (60), the $S\left(C^{r}\left(U_{i}\right)\right)$ stability of objective function component $C^{r}\left(U_{i}\right)$ is defined as

(61) $$S\left(C^{r}\left(U_{i}\right)\right)=\mathrm{sign}\left(\lambda \left(C^{r-1}\left(U_{i}\right),C^{r}\left(U_{i}\right),\tau^{r}\right)\right),$$

such that

(62) $$S\left(C^{r}\left(U_{i}\right)\right)=\left\{\begin{array}{c} S^{-}\left(C^{r}\left(U_{i}\right)\right),\;\mathrm{if}\;\mathrm{sign}\left(\lambda \left(C^{r-1}\left(U_{i}\right),C^{r}\left(U_{i}\right),\tau^{r}\right)\right)<0\\ S^{+}\left(C^{r}\left(U_{i}\right)\right),\;\mathrm{if}\;\mathrm{sign}\left(\lambda \left(C^{r-1}\left(U_{i}\right),C^{r}\left(U_{i}\right),\tau^{r}\right)\right)>0 \end{array}\right..$$

The function $S\left(C^{r}\left(U_{i}\right)\right)$ is stable if only

(63) $$S\left(C^{r}\left(U_{i}\right)\right)=S^{-}\left(C^{r}\left(U_{i}\right)\right),$$

and unstable as

(64) $$S\left(C^{r}\left(U_{i}\right)\right)=S^{+}\left(C^{r}\left(U_{i}\right)\right).$$

The proof is concluded here. □

**Figure 2:**
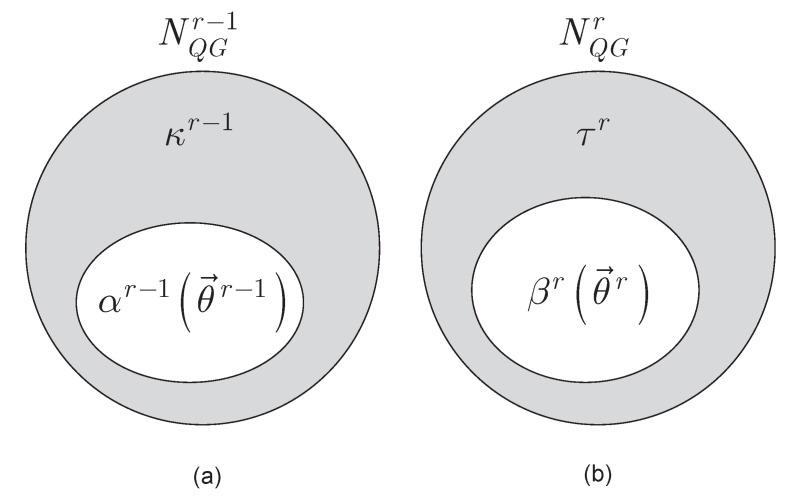
Components of the evolutionary model. (**a**) Model components in an $\left(r-1\right)$-th measurement round. For an $\left(r-1\right)$-th measurement round, vector $N_{QG}^{r-1}$ contains of the total $n_{QG}$, $n_{QG}\geq L$ objective function components, where $n_{QG}$ is the total number of unitary gates of the quantum computer and $N_{QG}^{r-1}={\left(C^{r-1}\left(U_{1}\right),\ldots ,C^{r-1}\left(U_{n_{QG}}\right)\right)}^{T}$, where $C^{r-1}\left(U_{i}\right)$ is an objective function component associated with an *i*-th unitary $U_{i}$ in an $\left(r-1\right)$-th measurement round, while $\kappa^{r-1}$ is defined as $\kappa^{r-1}=N_{QG}^{r-1}-\alpha^{r-1}\left({\vec{\theta }}^{r-1}\right)$, where $\alpha^{r-1}\left({\vec{\theta }}^{r-1}\right)$ is the objective function component vector at $U({\vec{\theta }}^{r-1})$, defined as $\alpha^{r-1}\left({\vec{\theta }}^{r-1}\right)={\left(C^{r-1}\left(U_{1}\right),\ldots ,C^{r-1}\left(U_{L}\right)\right)}^{T}$, while $U({\vec{\theta }}^{r-1})$ is a unitary sequence of an $\left(r-1\right)$-th measurement round, defined as $U\left({\vec{\theta }}^{r-1}\right)=U_{L}^{r-1}\left(\theta_{L}^{r-1}\right)U_{L-1}^{r-1}\left(\theta_{L-1}^{r-1}\right)\ldots U_{1}^{r-1}\left(\theta_{1}^{r-1}\right)$. (**b**) Model components in an *r*-th measurement round. The sets depict the ratio of the components. For an *r*-th measurement round, $N_{QG}^{r}$ contains of the total $n_{QG}$ objective function components as $N_{QG}^{r}={\left(C^{r}\left(U_{1}\right),\ldots ,C^{r}\left(U_{n_{QG}}\right)\right)}^{T}$, where $C^{r}\left(U_{i}\right)$ is an objective function component associated with an *i*-th unitary $U_{i}$ in an *r*-th measurement round, while $\tau^{r}$ is defined as $\tau^{r}=N_{QG}^{r}-\beta^{r1}\left({\vec{\theta }}^{r}\right)$, where $\beta^{r}\left({\vec{\theta }}^{r}\right)$ is the objective function component vector at $U({\vec{\theta }}^{r})$, $\beta^{r}\left({\vec{\theta }}^{r}\right)={\left(C^{r}\left(U_{1}\right),\ldots ,C^{r}\left(U_{L}\right)\right)}^{T}$, while $U({\vec{\theta }}^{r})$ is a unitary sequence of an *r*-th measurement round, $U\left({\vec{\theta }}^{r}\right)=U_{L}^{r}\left(\theta_{L}^{r}\right)U_{L-1}^{r}\left(\theta_{L-1}^{r}\right)\ldots U_{1}^{r}\left(\theta_{1}^{r}\right)$.

The main components of the evolutionary model are depicted in Figure 2.

A summary of the notations of the adaptive dynamics model is included in Table A2 of the Appendix A.

## 4. Canonical Equation

**Theorem** **2.** *(Canonical equation of adaptive objective function evaluation). An adaptive problem solving dynamics for a gate-model quantum computer is set via the canonical equation of objective function components.*

**Proof.**  Let $\alpha^{r-1}\left({\vec{\theta }}^{r-1}\right)$, $\tau^{r}$, $C^{r-1}\left(U_{i}\right)$, $C^{r}\left(U_{i}\right)$, and $\lambda \left(C^{r-1}\left(U_{i}\right),C^{r}\left(U_{i}\right),\tau^{r}\right)$ as defined in (33), (38), (43), (44) and (60), respectively. Then, by utilizing the framework of adaptive dynamics [39], the $f_{\varepsilon }\left(C^{r-1}\left(U_{i}\right)\right)$ evolution function (canonical equation [39,40,41]) of an objective function component $C^{r-1}\left(U_{i}\right)$ is defined as

(65) $$f_{\varepsilon }(C^{r-1}\left(U_{i}\right))=\frac{1}{2}\mu (C^{r-1})U_{i}()\sigma^{2}(C^{r-1}(U_{i})){\tilde{\alpha }}^{r-1}\left({\vec{\theta }}^{r-1}\right)X(C^{r}(U_{i})),$$

where

(66) $$\begin{array}{cc} X\left(C^{r}\left(U_{i}\right)\right) & =\frac{\partial }{\partial C^{r}\left(U_{i}\right)}\lambda {\left.\left(C^{r-1}\left(U_{i}\right),C^{r}\left(U_{i}\right),\tau^{r}\right)\right|}_{A=B}\\ \; & =\frac{\partial }{\partial C^{r}\left(U_{i}\right)}\lambda {\left.\left(C^{r-1}\left(U_{1}\right),C^{r-1}\left(U_{2}\right),\ldots ,C^{r-1}\left(U_{n_{QG}-L}\right),C^{r}\left(U_{i}\right),\tau^{r}\right)\right|}_{A=B}, \end{array}$$

while $\mu \left(C^{r-1}\left(U_{i}\right)\right)$ and $\sigma^{2}\left(C^{r-1}\left(U_{i}\right)\right)$ are derived as follows. Term $\mu \left(C^{r-1}\left(U_{i}\right)\right)$ is defined as a ratio of probabilities [39],

(67) $$\mu \left(C^{r-1}\left(U_{i}\right)\right)=\frac{{\mathrm{Pr}}^{*}\left(C^{r}\left(U_{i}\right)\neq C^{r-1}\left(U_{i}\right)\right)-\mathrm{Pr}\left(dt^{2}\right)}{\mathrm{Pr}\left(\tilde{S},C^{r-1}\left(U_{i}\right)>0\right)},$$

where ${\mathrm{Pr}}^{*}\left(C^{r}\left(U_{i}\right)\neq C^{r-1}\left(U_{i}\right)\right)$ is the probability of relation $C^{r}\left(U_{i}\right)\neq C^{r-1}\left(U_{i}\right)$ in a time interval $\left[t,t+dt\right]$, $\mathrm{Pr}\left(\tilde{S},C^{r-1}\left(U_{i}\right)>0\right)$ is the probability of a non-zero objective function component $C^{r-1}\left(U_{i}\right)$ in an $\left(r-1\right)$-th measurement round for a unitary $U_{i}$ at an equilibrium state $\tilde{S}$ (see (56)) [39], defined as

(68) $$\mathrm{Pr}\left(\tilde{S},C^{r-1}\left(U_{i}\right)>0\right)=f_{b}{\left({\tilde{\alpha }}^{r-1}\left({\vec{\theta }}^{r-1}\right),0,{\tilde{\kappa }}^{r-1},C^{r-1}\left(U_{i}\right),\cdot ,\tau^{r}\right)}^{r-1}{\tilde{\alpha }}^{r-1}\left({\vec{\theta }}^{r-1}\right)dt,$$

where $f_{b}\left(\cdot \right)$ is a rate function, while $\mathrm{Pr}\left(dt^{2}\right)$ is a probability [39] of that $C^{r}\left(U_{i}\right)\neq C^{r-1}\left(U_{i}\right)$ holds for more than one objective function components in an *r*-th measurement round.

The term $\sigma^{2}\left(C^{r-1}\left(U_{i}\right)\right)$ in (65) is defined as

(69) $$\begin{array}{cc} \; & \sigma^{2}\left(C^{r-1}\left(U_{i}\right)\right)=\left(E\left[{\left(C^{r}\left(U_{i}\right)-C^{r-1}\left(U_{i}\right)\right)}^{2}\right]\frac{1}{\varepsilon^{2}}\right)\\ \; & =\int_{-\infty }^{+\infty }{\left(\frac{\left(C^{r}\left(U_{i}\right)-C^{r-1}\left(U_{i}\right)\right)}{\varepsilon }\right)}^{2}D\left(C^{r-1}\left(U_{i}\right),\left(C^{r}\left(U_{i}\right)-C^{r-1}\left(U_{i}\right)\right)/\varepsilon \;\right)d\left(\frac{\left(C^{r}\left(U_{i}\right)-C^{r-1}\left(U_{i}\right)\right)}{\varepsilon }\right), \end{array}$$

where ε is a constant, such that a $F\left(C^{r-1}\left(U_{i}\right),C^{r}\left(U_{i}\right)-C^{r-1}\left(U_{i}\right)\right)$ distribution family is set as

(70) FCr−1Ui,CrUi−Cr−1Ui=DCr−1Ui,CrUi−Cr−1UiCrUi−Cr−1Uiεεε,

where $D\left(C^{r-1}\left(U_{i}\right),C^{r}\left(U_{i}\right)-C^{r-1}\left(U_{i}\right)\right)$ is a probability distribution with a standard deviation $\sigma =\sigma \left(C^{r-1}\left(U_{i}\right)\right)$, such that

(71) $$F\left(C^{r-1}\left(U_{i}\right),C^{r}\left(U_{i}\right)-C^{r-1}\left(U_{i}\right)\right)=F\left(C^{r-1}\left(U_{i}\right),C^{r-1}\left(U_{i}\right)-C^{r}\left(U_{i}\right)\right),$$

by theory.

Then, the role of $X\left(C^{r}\left(U_{i}\right)\right)$ from (66) can be interpreted as follows:

(72) $$X\left(C^{r}\left(U_{i}\right)\right)=\left\{\begin{array}{c} X\left(C^{r}\left(U_{i}\right)\right)\geq 0,\;\mathrm{if}\;\left|C^{r}\left(U_{i}\right)-C^{*}\left(U_{i}\right)\right|<\Delta_{\varepsilon }\\ X\left(C^{r}\left(U_{i}\right)\right)<0,\;\mathrm{if}\;\left|C^{r}\left(U_{i}\right)-C^{*}\left(U_{i}\right)\right|\geq \Delta_{\varepsilon } \end{array}\right.,$$

where $\Delta_{\varepsilon }$ is the distance between the objective function component $C^{r}\left(U_{i}\right)$ from a target value $C^{*}\left(U_{i}\right)$, from which a condition on the update mechanism of $C^{r-1}\left(U_{i}\right)$ to $C^{r}\left(U_{i}\right)$ can be defined as

(73) $$C^{r-1}\left(U_{i}\right)=\left\{\begin{array}{c} C^{r}\left(U_{i}\right),\;\mathrm{if}\;X\left(C^{r}\left(U_{i}\right)\right)\geq 0\;\\ C^{r-1}\left(U_{i}\right),\;\mathrm{if}\;X\left(C^{r}\left(U_{i}\right)\right)<0 \end{array}\right.,$$

since $C^{r-1}\left(U_{i}\right)$ updates to $C^{r}\left(U_{i}\right)$ only if $\left|C^{r}\left(U_{i}\right)-C^{*}\left(U_{i}\right)\right|<\Delta_{\varepsilon }$ holds, while $C^{r-1}\left(U_{i}\right)$ is not updated otherwise.

Since it is assumed that a greater value of the objective function component means that the objective function component is closer to the target value (since the aim is the maximization of a particular objective function of a computational problem fed in to the quantum computer), the conditions in (72) and (73) can be rewritten as follows:

(74) $$X\left(C^{r}\left(U_{i}\right)\right)=\left\{\begin{array}{c} X\left(C^{r}\left(U_{i}\right)\right)\geq 0,\;\mathrm{if}\;C^{r}\left(U_{i}\right)>C^{r-1}\left(U_{i}\right)\\ X\left(C^{r}\left(U_{i}\right)\right)<0,\;\mathrm{if}\;C^{r}\left(U_{i}\right)\leq C^{r-1}\left(U_{i}\right) \end{array}\right.,$$

and

(75) $$C^{r-1}\left(U_{i}\right)=\left\{\begin{array}{c} C^{r}\left(U_{i}\right),\;\mathrm{if}\;C^{r}\left(U_{i}\right)>C^{r-1}\left(U_{i}\right)\;\\ C^{r-1}\left(U_{i}\right),\;\mathrm{if}\;C^{r}\left(U_{i}\right)\leq C^{r-1}\left(U_{i}\right) \end{array}\right.,$$

respectively.

By using the notation $C^{r-1}\left(U_{i}\left(t\right)\right)$ for the objective function component value at a particular *t*, the result in (65) can also be rewritten as [39]

(76) $$f_{\varepsilon }\left(C^{r-1}\left(U_{i}\right)\right)=\lim_{dt\to 0}\frac{E\left[C^{r-1}\left(U_{i}\left(t+dt\right)\right)-C^{r-1}\left(U_{i}\left(t\right)\right)\right]}{dt},$$

where *t* is between $t\in \left[0,T\right]$, where *T* is the total evolution time of the quantum computer.

Let us assume that the relation of $C^{r}\left(U_{i}\right)>C^{r-1}\left(U_{i}\right)$ holds with probability $\mathrm{Pr}(C^{r}\left(U_{i}\right)>$$C^{r-1}\left(U_{i}\right))$, defined at some constant ζ, as

(77) $$\mathrm{Pr}\left(C^{r}\left(U_{i}\right)>C^{r-1}\left(U_{i}\right)\right)=\left\{\begin{array}{c} \frac{\lambda \left(C^{r-1}\left(U_{i}\right),C^{r}\left(U_{i}\right),\tau^{r}\right)}{\zeta },\;\mathrm{if}\;X\left(C^{r}\left(U_{i}\right)\right)\left|C^{r}\left(U_{i}\right)-C^{r-1}\left(U_{i}\right)\right|>0\\ 0,\;\mathrm{otherwise} \end{array}\right.,$$

while $\mathrm{Pr}\left(C^{r}\left(U_{i}\right),C^{r}\left(U_{i}\right)+dC^{r}\left(U_{i}\right)\right)$ is the probability that $C^{r}\left(U_{i}\right)$ is in the interval of

$\left[C^{r}\left(U_{i}\right),C^{r}\left(U_{i}\right)+dC^{r}\left(U_{i}\right)\right]$, defined via (70) at a particular ε, as

(78) $$\begin{array}{cc} \; & \mathrm{Pr}\left(C^{r}\left(U_{i}\right),C^{r}\left(U_{i}\right)+dC^{r}\left(U_{i}\right)\right)\\ \; & =F\left(C^{r-1}\left(U_{i}\right),C^{r}\left(U_{i}\right)-C^{r-1}\left(U_{i}\right)\right)\\ \; & =\frac{D\left(C^{r-1}\left(U_{i}\right),\left(C^{r}\left(U_{i}\right)-C^{r-1}\left(U_{i}\right)\right)/\varepsilon \;\right)}{\varepsilon }. \end{array}$$

Then, (76) can be evaluated as

(79) $$f_{\varepsilon }\left(C^{r-1}\left(U_{i}\right)\right)=\lim_{dt\to 0}\frac{1}{dt}\int_{-\infty }^{+\infty }\left(C^{r}\left(U_{i}\right)-C^{r-1}\left(U_{i}\right)\varphi \left(C^{r-1}\left(U_{i}\right)\right)dC^{r}\left(U_{i}\right)\right),$$

where $\varphi \left(C^{r-1}\left(U_{i}\right)\right)$ is a probability that the objective function component is updated from $C^{r-1}\left(U_{i}\right)$ to $C^{r}\left(U_{i}\right)$ such that the value of $C^{r}\left(U_{i}\right)$ at an time interval $\left[t,t+dt\right]$ is in the interval of $\left[C^{r}\left(U_{i}\right),C^{r}\left(U_{i}\right)+dC^{r}\left(U_{i}\right)\right]$, defined as

(80) $$\begin{array}{cc} \; & \varphi \left(C^{r-1}\left(U_{i}\right)\right)\\ \; & ={\mathrm{Pr}}^{*}\left(C^{r}\left(U_{i}\right)\neq C^{r-1}\left(U_{i}\right)\right)\cdot \mathrm{Pr}\left(C^{r}\left(U_{i}\right)>C^{r-1}\left(U_{i}\right)\right)\cdot \mathrm{Pr}\left(C^{r}\left(U_{i}\right),C^{r}\left(U_{i}\right)+dC^{r}\left(U_{i}\right)\right), \end{array}$$

where ${\mathrm{Pr}}^{*}\left(C^{r}\left(U_{i}\right)\neq C^{r-1}\left(U_{i}\right)\right)$ is as used in (67).

After some calculations, the term $\varphi \left(C^{r-1}\left(U_{i}\right)\right)dC^{r}\left(U_{i}\right)$ in (79) can be evaluated in a closed-form [39], as

(81) $$\begin{array}{cc} \; & \varphi \left(C^{r-1}\left(U_{i}\right)\right)dC^{r}\left(U_{i}\right)dC^{r}\left(U_{i}\right)\\ \; & =\mu \left(C^{r-1}\left(U_{i}\right)\right)\zeta \alpha^{r-1}({\vec{\theta }}^{r-1})\mathrm{Pr}\left(C^{r}\left(U_{i}\right)>C^{r-1}\left(U_{i}\right)\right)F\left(C^{r-1}\left(U_{i}\right),C^{r}\left(U_{i}\right)-C^{r-1}\left(U_{i}\right)\right)dC^{r}\left(U_{i}\right)dt, \end{array}$$

where $\mu \left(C^{r-1}\left(U_{i}\right)\right)$ is defined in (67), $\alpha^{r-1}\left({\vec{\theta }}^{r-1}\right)$ is given in (33), $F(C^{r-1}\left(U_{i}\right),C^{r}\left(U_{i}\right)-$$C^{r-1}\left(U_{i}\right))$ is defined in (70), while $\mathrm{Pr}\left(C^{r}\left(U_{i}\right)>C^{r-1}\left(U_{i}\right)\right)$ is given in (77).

Then, by putting (81) into (79), after some additional steps leads to (65) from (79), the probability functions of (80) determine the evolution of a particular objective function. The adaptive problem solving dynamics in a gate-model quantum computer is therefore characterized via the evolution function $f_{\varepsilon }\left(C^{r-1}\left(U_{i}\right)\right)$, which function identifies a canonical equation.

The proof is concluded here. □

## 5. Superposition of Stability Functions

**Theorem** **3.** *(Superposition stability functions). In the computational stage, the objective function stabilities formulate a superposition in a gate-model quantum computer.*

**Proof.**  Let *A* and *B* as defined in (43) and (44), and let us assume that

(82) $$\bar{X}={\bar{X}}_{i}^{r-1}={\bar{X}}_{i}^{r},$$

and

(83) $$\rho =\rho_{i}^{r-1}=\rho_{i}^{r},$$

respectively.

Then, let $\lambda \left(A,B,W\right)$ be the fitness function, as

(84) $$\begin{array}{cc} \lambda \left(A,B,W\right)= & \frac{1}{2}\Psi \left(C^{r-1}\left(U_{i}\right)\right){\left(A-\bar{X}\right)}^{2}\\ \; & +\frac{\partial^{2}}{\partial A\partial B}\lambda {\left.\left(A,B,W\right)\right|}_{A=B=\bar{X}}\left(A-\bar{X}\right)\left(B-\bar{X}\right)\\ \; & +\frac{1}{2}\Psi \left(C^{r}\left(U_{i}\right)\right){\left(B-\bar{X}\right)}^{2}+O\left({∥\left(A-\bar{X},B-\bar{X}\right)∥}^{3}\right), \end{array}$$

where *W* refers to $\tau^{r}$ in an equilibrium state, as

(85) $$W={\tilde{\tau }}^{r},$$

while $\Psi \left(C^{r-1}\left(U_{i}\right)\right)$ is defined as

(86) $$\Psi \left(C^{r-1}\left(U_{i}\right)\right)=\frac{\partial^{2}}{\partial A^{2}}\lambda {\left.\left(A,\bar{X},W\right)\right|}_{A=\bar{X}},$$

while $\Psi \left(C^{r}\left(U_{i}\right)\right)$ is defined as

(87) $$\Psi \left(C^{r}\left(U_{i}\right)\right)=\frac{\partial^{2}}{\partial B^{2}}\lambda {\left.\left(\bar{X},B,W\right)\right|}_{B=\bar{X}},$$

and

(88) $$\left(\frac{\partial }{\partial A}\lambda \left(A,A,W\right)=\tilde{X}\left(C^{r-1}\left(U_{i}\right)\right)\right)+\left(\tilde{X}\left(C^{r}\left(U_{i}\right)\right)\right)=0.$$

where

(89) $$\tilde{X}\left(C^{r-1}\left(U_{i}\right)\right)=\frac{\partial }{\partial A}\lambda {\left.\left(A,B,W\right)\right|}_{B=A},$$

and

(90) $$\tilde{X}\left(C^{r}\left(U_{i}\right)\right)=\frac{\partial }{\partial B}\lambda {\left.\left(A,B,W\right)\right|}_{B=A}.$$

Using (43) and (44), the result in (84) can be rewritten as

(91) $$\begin{array}{cc} \lambda \left(A,B,W\right)= & \frac{\rho^{2}}{2}\left(\Psi \right.\left(C^{r-1}\left(U_{i}\right)\right)\cos^{2}\left(\theta_{i}^{r}\right)\\ \; & +2\frac{\partial^{2}}{\partial A\partial B}\lambda {\left.\left(A,B,W\right)\right|}_{A=B=\bar{X}}\cos\left(\theta_{i}^{r}\right)\sin\left(\theta_{i}^{r}\right)\\ \; & +\Psi \left(C^{r}\left(U_{i}\right)\right)\left.\sin^{2}\left(\theta_{i}^{r}\right)\right)+O\left(\rho^{3}\right). \end{array}$$

with relation

(92) $$\begin{array}{cc} \left(\frac{\partial^{2}}{\partial A^{2}}\lambda \left(A,A,W\right)=\frac{\partial^{2}}{\partial A^{2}}\lambda {\left.\left(A,B,W\right)\right|}_{B=A}\right) & \\ +2\frac{\partial^{2}}{\partial A\partial B}\lambda {\left.\left(A,B,W\right)\right|}_{B=A} & \\ +\frac{\partial^{2}}{\partial B^{2}}\lambda {\left.\left(A,B,W\right)\right|}_{B=A} & \\ =0, & \end{array}$$

where

(93) $$2\frac{\partial^{2}}{\partial A\partial B}\lambda {\left.\left(A,B,W\right)\right|}_{B=A}=-\frac{\partial^{2}}{\partial A^{2}}\lambda {\left.\left(A,B,W\right)\right|}_{B=A}-\frac{\partial^{2}}{\partial BA^{2}}\lambda {\left.\left(A,B,W\right)\right|}_{B=A}.$$

Then, by utilizing (92) and (93), (91) can be simplified as

(94) $$\begin{array}{cc} \lambda \left(A,B,W\right)= & \Psi \left(C^{r-1}\left(U_{i}\right)\right)\cos\left(\theta_{i}^{r}\right)-\Psi \left(C^{r}\left(U_{i}\right)\right)\left.\sin\left(\theta_{i}^{r}\right)\right)\\ \; & \cdot \frac{\rho^{2}}{2}\left(\cos\left(\theta_{i}^{r}\right)-\sin\left(\theta_{i}^{r}\right)\right)+O\left(\rho^{3}\right). \end{array}$$

The function $\lambda \left(A,B,W\right)$ in (94) changes sign if

(95) $$\theta_{i}^{r}\in \left\{\frac{\pi }{4},\zeta_{i}^{r}\right\},$$

where $\zeta_{i}^{r}$ is a gate parameter value, defined as

(96) $$\zeta_{i}^{r}=\tan^{-1}\left(\frac{\Psi \left(C^{r-1}\left(U_{i}\right)\right)}{\Psi \left(C^{r}\left(U_{i}\right)\right)}\right).$$

For a given $C^{r}\left(U_{i}\right)$, the $S\left(C^{r}\left(U_{i}\right)\right)$ stability function is defined as

(97) $$S\left(C^{r}\left(U_{i}\right)\right)=\mathrm{sign}\left(\lambda \left(A,B,W\right)\right),$$

such that $S\left(C^{r}\left(U_{i}\right)\right)$ is stable if $S\left(C^{r}\left(U_{i}\right)\right)<0$, and unstable otherwise, denoted by

(98) $$S\left(C^{r}\left(U_{i}\right)\right)=\left\{\begin{array}{c} S^{-}\left(C^{r}\left(U_{i}\right)\right),\;\mathrm{if}\;\mathrm{sign}\left(\lambda \left(A,B,W\right)\right)<0\\ S^{+}\left(C^{r}\left(U_{i}\right)\right),\;\mathrm{if}\;\mathrm{sign}\left(\lambda \left(A,B,W\right)\right)>0 \end{array}\right..$$

In the computational procedure, both outcomes of $S\left(C^{r}\left(U_{i}\right)\right)$ exist in parallel in the quantum computer for a given $\theta_{i}^{r}$; thus, the stability functions formulate a superposition $S^{\prime }\left(C^{r}\left(U_{i}\right)\right)$ with respect to a particular objective function component $C^{r}\left(U_{i}\right)$, as

(99) $$S^{\prime }\left(C^{r}\left(U_{i}\right)\right)=pS^{+}\left(C^{r}\left(U_{i}\right)\right)+\left(1-p\right)S^{-}\left(C^{r}\left(U_{i}\right)\right),$$

where *p* is the probability of an unstable stability function $S^{+}\left(C^{r}\left(U_{i}\right)\right)$. The *M* measurement sets $S^{\prime }\left(C^{r}\left(U_{i}\right)\right)$ to a determined value (stable or unstable) according to (99).

It can be straightforwardly verified that, for the components of the superposed $S^{\prime }\left(C^{r}\left(U_{i}\right)\right)$, the sum of fitness function derivatives is

(100) $$S^{\prime }\left(C^{r}\left(U_{i}\right)\right)=pF\left(S^{+}\left(C^{r}\left(U_{i}\right)\right)\right)+\left(1-p\right)F\left(S^{-}\left(C^{r}\left(U_{i}\right)\right)\right),$$

where function $F\left(S^{-}\left(C^{r}\left(U_{i}\right)\right)\right)$ is defined for the stable components, as

(101) $$F\left(S^{-}\left(C^{r}\left(U_{i}\right)\right)\right)=\Psi \left(C^{r-1}\left(U_{i}\right)\right)+\Psi \left(C^{r}\left(U_{i}\right)\right)<0,$$

while function $F\left(S^{+}\left(C^{r}\left(U_{i}\right)\right)\right)$ is defined for the for the unstable components, as

(102) $$F\left(S^{+}\left(C^{r}\left(U_{i}\right)\right)\right)=\Psi \left(C^{r-1}\left(U_{i}\right)\right)+\Psi \left(C^{r}\left(U_{i}\right)\right)>0.$$

Then, using (93) with (101) yields

(103) $$F\left(S^{-}\left(C^{r}\left(U_{i}\right)\right)\right)=\frac{\partial^{2}}{\partial A\partial B}\lambda {\left.\left(A,B,W\right)\right|}_{A=B=\bar{X}}>0,$$

while (93) with (102) yields

(104) $$F\left(S^{+}\left(C^{r}\left(U_{i}\right)\right)\right)=\frac{\partial^{2}}{\partial A\partial B}\lambda {\left.\left(A,B,W\right)\right|}_{A=B=\bar{X}}<0.$$

Then, the superposition in (100) can be rewritten:

(105) $$S^{\prime }\left(C^{r}\left(U_{i}\right)\right)=p\left(\frac{\partial^{2}}{\partial A\partial B}\lambda {\left.\left(A,B,W\right)\right|}_{A=B=\bar{X}}>0\right)+\left(1-p\right)\left(\frac{\partial^{2}}{\partial A\partial B}\lambda {\left.\left(A,B,W\right)\right|}_{A=B=\bar{X}}<0\right),$$

Since the value of $\lambda \left(A,B,W\right)$ in (94) depends on the gate parameter $\theta_{i}^{r}$, the stability in (99) can be satisfied via the selection of $\theta_{i}^{r}$, such that $\mathrm{sign}\left(\lambda \left(A,B,W\right)\right)<0$ holds, which results in a stable objective function in an *r*-th iteration with a unit probability in $S^{\prime }\left(C^{r}\left(U_{i}\right)\right)$, p=0,

(106) $$S^{\prime }\left(C^{r}\left(U_{i}\right)\right)=\left(\frac{\partial^{2}}{\partial A\partial B}\lambda {\left.\left(A,B,W\right)\right|}_{A=B=\bar{X}}<0\right)=S^{-}\left(C^{r}\left(U_{i}\right)\right),$$

As follows, the gate parameters of the unitaries can be set such that a *M* measurement results stable components with a probability

(107) $$\begin{array}{cc} p_{suc}^{M}\left(S^{\prime }\left(C^{r}\left(U_{i}\right)\right)=S^{-}\left(C^{r}\left(U_{i}\right)\right)\right) & =\left(1-p\right)\left(1-p_{i}^{r}\right)\\ \; & =\sin^{2}\left(\theta_{i}^{r}\right), \end{array}$$

where $p_{i}^{r}$ is given in (40).

The proof is concluded here. □

**Figure 3:**
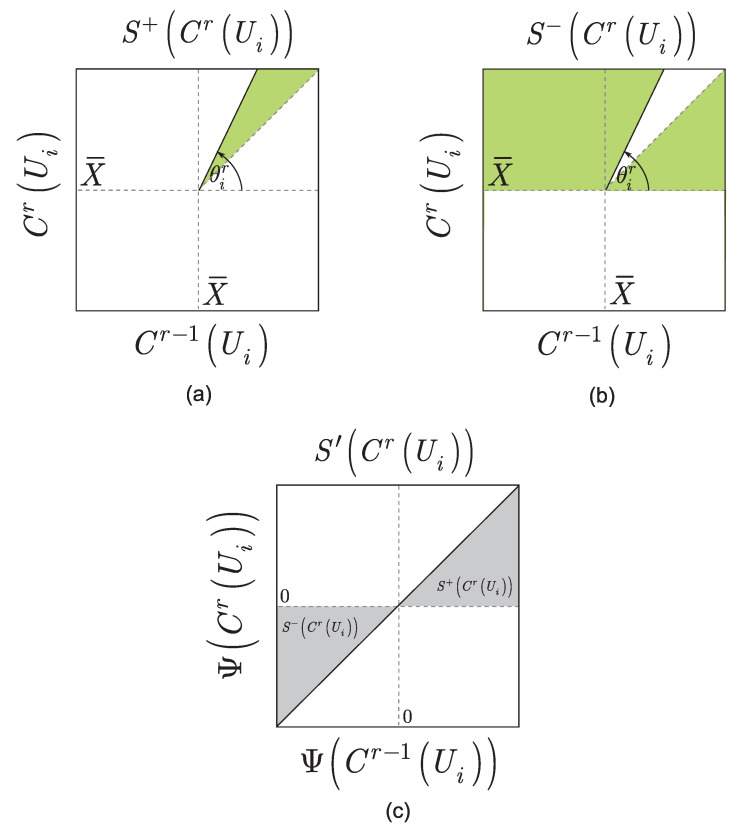
(**a**) Stability function $S\left(C^{r}\left(U_{i}\right)\right)$ of an unstable objective function component $C^{r}\left(U_{i}\right)$ in the space of $\left\{C^{r-1}\left(U_{i}\right),C^{r}\left(U_{i}\right)\right\}$, $C^{r-1}\left(U_{i}\right)={\bar{X}}_{i}^{r-1}+\rho_{i}^{r-1}\cos\left(\theta_{i}^{r}\right)$, $C^{r}\left(U_{i}\right)={\bar{X}}_{i}^{r}+\rho_{i}^{r}\sin\left(\theta_{i}^{r}\right)$, $\theta_{i}^{r}\in \left[0,\pi \right]$ is the gate parameter of a unitary $U_{i}$ in an *r*-th measurement round; $C^{r}\left(U_{i}\right)$ is the objective function component associated with $U_{i}$ in an *r*-th measurement round; and ρ and $\bar{X}$ are constants, set as $\bar{X}={\bar{X}}_{i}^{r-1}={\bar{X}}_{i}^{r}$, $\rho =\rho_{i}^{r-1}=\rho_{i}^{r}$. The points $\left(C^{r-1}\left(U_{i}\right),C^{r}\left(U_{i}\right)\right)$ are dominate in the white region, where the stability function is positive, $S\left(C^{r}\left(U_{i}\right)\right)=S^{+}\left(C^{r}\left(U_{i}\right)\right)$, while it is negative in the green region. (**b**) Stability function $S\left(C^{r}\left(U_{i}\right)\right)$ of a stable objective function component $C^{r}\left(U_{i}\right)$ in the space of $\left\{C^{r-1}\left(U_{i}\right),C^{r}\left(U_{i}\right)\right\}$. The points $\left(C^{r-1}\left(U_{i}\right),C^{r}\left(U_{i}\right)\right)$ dominate in the green region, where the stability function is negative, $S\left(C^{r}\left(U_{i}\right)\right)=S^{-}\left(C^{r}\left(U_{i}\right)\right)$, and $\bar{X}$ is a constant. (**c**) Superposition $S^{\prime }\left(C^{r}\left(U_{i}\right)\right)=pS^{+}\left(C^{r}\left(U_{i}\right)\right)+\left(1-p\right)S^{-}\left(C^{r}\left(U_{i}\right)\right)$ of the stability functions $S^{+}\left(C^{r}\left(U_{i}\right)\right)$ and $S^{-}\left(C^{r}\left(U_{i}\right)\right)$ (gray regions) in the quantum computer before a measurement, in the space of $\left\{\Psi \left(C^{r-1}\left(U_{i}\right)\right),\Psi \left(C^{r}\left(U_{i}\right)\right)\right\}$, $\Psi \left(C^{r-1}\left(U_{i}\right)\right)=\frac{\partial^{2}}{\partial A^{2}}\lambda {\left.\left(A,\bar{X},W\right)\right|}_{A=\bar{X}}$, and $\Psi \left(C^{r}\left(U_{i}\right)\right)=\frac{\partial^{2}}{\partial B^{2}}\lambda {\left.\left(\bar{X},B,W\right)\right|}_{B=\bar{X}}$.

The $S^{\prime }\left(C^{r}\left(U_{i}\right)\right)$ superposition of the objective function stability functions for a particular $\theta_{i}^{r}$ is depicted in Figure 3.

## 6. Conclusions

Here, we defined a mathematical model of adaptive problem solving dynamics in gate-model quantum computers. The objective function to be maximized by the gate-model quantum computer is associated with an optimization problem. We characterized a canonical equation of adaptive problem solving dynamics. As future work, our aim is to implement the proposed model with particular input problems to verify the theoretical results.

## Data Availability

This work does not have any experimental data.

## Appendix A

### Appendix A.1. Abbreviations

**AD** Adaptive Dynamics

**QAOA** Quantum Approximate Optimization Algorithm

### Appendix A.2. Notations

The notations of the manuscript are summarized in Table A1.

**Table A1:** Summary of notations.

Notation	Description
$U_{i}\left(\theta_{i}\right)$	An *i*-th unitary gate, $U_{i}\left(\theta_{i}\right)=\exp\left(-i\theta_{i}P\right)$, where *P* is a generalized Pauli operator formulated by a tensor product of Pauli operators $\left\{X,Y,Z\right\}$, while $\theta_{i}$ is the gate parameter associated with $U_{i}\left(\theta_{i}\right)$.
$\theta_{i}$	Gate parameter of $U_{i}$.
$U(\vec{\theta })$	System state, $U\left(\vec{\theta }\right)=U_{L}\left(\theta_{L}\right)U_{L-1}\left(\theta_{L-1}\right)\ldots U_{1}\left(\theta_{1}\right)$, where $U_{i}\left(\theta_{i}\right)$ identifies an *i*-th unitary gate.
$\vec{\theta }$	Gate parameter vector of the *L* unitaries, $\vec{\theta }={\left(\theta_{1},\ldots ,\theta_{L}\right)}^{T}$.
$C\left(z\right)$	Classical objective function of a computational problem fed into the quantum computer, $C\left(z\right)=\sum_{\forall \langle ij\rangle }C_{\langle ij\rangle }\left(z\right)$, where $C_{\langle ij\rangle }\left(z\right)$ is an objective function component evaluated between quantum qubits ij in the QG structure of the gate-model quantum computer.
*L*	Number of unitaries of the quantum computer.
*n*	Number of qubits of input state $|s\rangle$; bit length of string *z*.
$n_{QG}$	Total number of unitary gates of the quantum computer.
*X*	Pauli *X* operator.
*Z*	Pauli *Z* operator.
*Y*	Pauli *Y* operator.
*L*	Number of unitaries in a particular unitary sequence.
*R*	Number of total measurement rounds set for the optimization problem fed into the quantum computer.
$C^{*}$	Optimal (target) objective function value.
*r*	An *r*-th measurement round, r=1,…,R.
$z^{r}$	A string resulting from a measurement in an *r*-th measurement round, r=1,…,R.
$C\left(z^{r}\right)$	Objective function evaluated via a string $z^{r}$ of an *r*-th measurement round, r=1,…,R.
$C_{\langle ij\rangle }^{r}\left(z^{r}\right)$	Objective function component in $C\left(z^{r}\right)$ associated with a connection between unitaries $U_{i}$ and $U_{j}$.
$z_{i}^{r}$	An *i*-th bit of $z^{r}$, $z_{i}^{r}\in \left\{-1,1\right\}$.
$C_{i}^{r}$	Objective function component defined for $U_{i}$ in an *r*-th measurement round.
$C_{j}^{r}$	Objective function component defined for $U_{j}$ in an *r*-th measurement round.
$\theta_{i}^{r}$	Gate parameter of unitary $U_{i}$ in an *r*-th measurement round.
$C^{r}\left(U_{i}\right)$	Objective function component associated with $U_{i}$ in an *r*-th measurement round
$f\left(C^{r}\left(U_{i}\right)\right)$	Objective function update function, $f\left(C^{r}\left(U_{i}\right)\right)=p_{i}^{r}C^{r-1}\left(U_{i}\right)+\left(1-p_{i}^{r}\right)C^{r}\left(U_{i}\right),$ where $C^{r-1}\left(U_{i}\right)$ is the objective function component at $U_{i}=I$, while $C^{r}\left(U_{i}\right)$ is an updated objective function component if $U_{i}\neq I$.
*A*	$A=C^{r-1}\left(U_{i}\right)={\bar{X}}_{i}^{r-1}+\rho_{i}^{r-1}\cos\left(\theta_{i}^{r}\right)$, where $\bar{X}$ and ρ are some constants.
*B*	$B=C^{r}\left(U_{i}\right)={\bar{X}}_{i}^{r}+\rho_{i}^{r}\sin\left(\theta_{i}^{r}\right)$, where $\bar{X}$ and ρ are some constants.
$C^{*}$	Target objective function value subject to be reached in *R* measurement rounds.
$C_{i}^{*}$	Target objective function component set for $U_{i}$.
$U({\vec{\theta }}^{r-1})$	A unitary sequence of an $\left(r-1\right)$-th measurement round, $U\left({\vec{\theta }}^{r-1}\right)=U_{L}^{r-1}\left(\theta_{L}^{r-1}\right)U_{L-1}^{r-1}\left(\theta_{L-1}^{r-1}\right)\ldots U_{1}^{r-1}\left(\theta_{1}^{r-1}\right)$.
$\alpha^{r-1}\left({\vec{\theta }}^{r-1}\right)$	Objective function component vector at $U({\vec{\theta }}^{r-1})$, $\alpha^{r-1}\left({\vec{\theta }}^{r-1}\right)={\left(C^{r-1}\left(U_{1}\right),\ldots ,C^{r-1}\left(U_{L}\right)\right)}^{T}$.
$\beta^{r}\left({\vec{\theta }}^{r}\right)$	Objective function component vector at $U({\vec{\theta }}^{r})$, $\beta^{r}\left({\vec{\theta }}^{r}\right)={\left(C^{r}\left(U_{1}\right),\ldots ,C^{r}\left(U_{L}\right)\right)}^{T}$.
$N_{QG}^{r-1}$	Vector of the total $n_{QG}$, $n_{QG}\geq L$ objective function components at an $\left(r-1\right)$-th measurement round, $N_{QG}^{r-1}={\left(C^{r-1}\left(U_{1}\right),\ldots ,C^{r-1}\left(U_{n_{QG}}\right)\right)}^{T}.$
$\kappa^{r-1}$	A vector in an $\left(r-1\right)$-th measurement round, defined as $\kappa^{r-1}=N_{QG}^{r-1}-\alpha^{r-1}\left({\vec{\theta }}^{r-1}\right)$.
$\tau^{r}$	An *r*-th measurement round, $\tau^{r}=N_{QG}^{r}-\beta^{r}\left({\vec{\theta }}^{r}\right)$.
$J\left(C^{r-1}\left(U_{i}\right),\tau^{r}\right)$	A Jacobian matrix at a particular $C^{r-1}\left(U_{i}\right)$ and $\tau^{r}$.
${\tilde{\alpha }}^{r-1}\left({\vec{\theta }}^{r-1}\right)$	A vector identifying $\alpha^{r-1}\left({\vec{\theta }}^{r-1}\right)$ at an equilibrium state.
${\tilde{\kappa }}^{r-1}$	A vector referring to vector $\kappa^{r-1}$ at an equilibrium state.
$f\left(\cdot \right)$	A function evaluating the $\alpha^{r-1}{\left({\vec{\theta }}^{r-1}\right)}^{\prime }$ derivative of $\alpha^{r-1}\left({\vec{\theta }}^{r-1}\right)$.
$F\left(\cdot \right)$	A function evaluating the ${\kappa^{r-1}}^{\prime }$ derivative of $\kappa^{r-1}$.
$\lambda_{1}$	A coefficient identifying the number of non-zero $C^{r-1}\left(U_{1},\ldots ,U_{L}\right)$ objective function components in an $\left(r-1\right)$-th measurement round taken over unitaries $U_{1},\ldots ,U_{L}$.
$\lambda_{2}$	A coefficient referring to the number of zero $C^{r-1}\left(U_{1},\ldots ,U_{L}\right)$ objective function components of an $\left(r-1\right)$-th measurement round taken over $U_{1},\ldots ,U_{L}$.
S	State space, $S≐(\alpha^{r-1}\left({\vec{\theta }}^{r-1}\right),\beta^{r}\left({\vec{\theta }}^{r}\right),\kappa^{r-1})$.
$\tilde{S}$	Equilibrium state $\tilde{S}$, $\tilde{S}≐({\tilde{\alpha }}^{r-1}\left({\vec{\theta }}^{r-1}\right),0,{\tilde{\kappa }}^{r-1})$.
$J_{\tilde{S}}\left(\cdot \right)$	Jacobian matrix at the equilibrium $\tilde{S}$, $J_{\tilde{S}}\left(C^{r-1}\left(U_{i}\right),C^{r}\left(U_{i}\right),\tau^{r}\right)$.
$\lambda \left(\cdot \right)$	An eigenvalue of $J_{\tilde{S}}\left(C^{r-1}\left(U_{i}\right),C^{r}\left(U_{i}\right),\tau^{r}\right)$, $\lambda \left(C^{r-1}\left(U_{i}\right),C^{r}\left(U_{i}\right),\tau^{r}\right)$.
$S\left(C^{r}\left(U_{i}\right)\right)$	Stability of objective function component $C^{r}\left(U_{i}\right)$, $S\left(C^{r}\left(U_{i}\right)\right)=\mathrm{sign}\left(\lambda \left(C^{r-1}\left(U_{i}\right),C^{r}\left(U_{i}\right),\tau^{r}\right)\right)$.
$S^{-}\left(C^{r}\left(U_{i}\right)\right)$	A stable $S\left(C^{r}\left(U_{i}\right)\right)$ function.
$S^{+}\left(C^{r}\left(U_{i}\right)\right)$	An unstable $S\left(C^{r}\left(U_{i}\right)\right)$ function.
$f_{\varepsilon }\left(C^{r-1}\left(U_{i}\right)\right)$	Evolution function (canonical equation) of an objective function component $C^{r-1}\left(U_{i}\right)$.
$X\left(C^{r}\left(U_{i}\right)\right)$	Coefficient defined for $C^{r}\left(U_{i}\right)$, $X\left(C^{r}\left(U_{i}\right)\right)=\frac{\partial }{\partial C^{r}\left(U_{i}\right)}\lambda {\left.\left(C^{r-1}\left(U_{i}\right),C^{r}\left(U_{i}\right),\tau^{r}\right)\right|}_{A=B}.$
$\mu \left(C^{r-1}\left(U_{i}\right)\right)$	A ratio of probabilities.
$f_{b}\left(\cdot \right)$	A rate function.
$\sigma^{2}\left(C^{r-1}\left(U_{i}\right)\right)$	A term in the canonical equation.
ε	A constant.
$F\left(\cdot \right)$	A distribution family, $F\left(C^{r-1}\left(U_{i}\right),C^{r}\left(U_{i}\right)-C^{r-1}\left(U_{i}\right)\right)$.
$D\left(\cdot \right)$	A probability distribution with a standard deviation $\sigma =\sigma \left(C^{r-1}\left(U_{i}\right)\right)$, $D\left(C^{r-1}\left(U_{i}\right),C^{r}\left(U_{i}\right)-C^{r-1}\left(U_{i}\right)\right)$.
$\Delta_{\varepsilon }$	Distance between the objective function component $C^{r}\left(U_{i}\right)$ from a target value $C^{*}\left(U_{i}\right)$.
ζ	A constant.
$\varphi \left(C^{r-1}\left(U_{i}\right)\right)$	Probability that the objective function component is updated from $C^{r-1}\left(U_{i}\right)$ to $C^{r}\left(U_{i}\right)$ such that the value of $C^{r}\left(U_{i}\right)$ at an time interval $\left[t,t+dt\right]$ is in the interval of $\left[C^{r}\left(U_{i}\right),C^{r}\left(U_{i}\right)+dC^{r}\left(U_{i}\right)\right]$.
*W*	A vector referring to $\tau^{r}$ in an equilibrium state as $W={\tilde{\tau }}^{r}$.
$\Psi \left(C^{r-1}\left(U_{i}\right)\right)$	Coefficient for $C^{r-1}\left(U_{i}\right)$, $\Psi \left(C^{r-1}\left(U_{i}\right)\right)=\frac{\partial^{2}}{\partial A^{2}}\lambda {\left.\left(A,\bar{X},W\right)\right|}_{A=\bar{X}}$.
$\Psi \left(C^{r}\left(U_{i}\right)\right)$	Coefficient for $C^{r}\left(U_{i}\right)$, $\Psi \left(C^{r}\left(U_{i}\right)\right)=\frac{\partial^{2}}{\partial B^{2}}\lambda {\left.\left(\bar{X},B,W\right)\right|}_{B=\bar{X}}$.
$\tilde{X}\left(C^{r-1}\left(U_{i}\right)\right)$	Coefficient for $C^{r-1}\left(U_{i}\right)$ at an equilibrium, $\tilde{X}\left(C^{r-1}\left(U_{i}\right)\right)=\frac{\partial }{\partial A}\lambda {\left.\left(A,B,W\right)\right|}_{B=A}$.
$\tilde{X}\left(C^{r}\left(U_{i}\right)\right)$	Coefficient for $C^{r}\left(U_{i}\right)$ at an equilibrium, $\tilde{X}\left(C^{r}\left(U_{i}\right)\right)=\frac{\partial }{\partial B}\lambda {\left.\left(A,B,W\right)\right|}_{B=A}$.
$\zeta_{i}^{r}$	A gate parameter value defined for a unitary $U_{i}$ in an *r*-th measurement round.
$S^{\prime }\left(C^{r}\left(U_{i}\right)\right)$	Superposition of stability functions with respect to a particular objective function component $C^{r}\left(U_{i}\right)$, $S^{\prime }\left(C^{r}\left(U_{i}\right)\right)=pS^{+}\left(C^{r}\left(U_{i}\right)\right)+\left(1-p\right)S^{-}\left(C^{r}\left(U_{i}\right)\right)$, where *p* is the probability of an unstable stability function $S^{+}\left(C^{r}\left(U_{i}\right)\right)$.
$F\left(S^{+}\left(C^{r}\left(U_{i}\right)\right)\right)$	A function, defined for the for the $S^{+}\left(C^{r}\left(U_{i}\right)\right)$ unstable component, $F\left(S^{+}\left(C^{r}\left(U_{i}\right)\right)\right)=\Psi \left(C^{r-1}\left(U_{i}\right)\right)+\Psi \left(C^{r}\left(U_{i}\right)\right)>0$.
$F\left(S^{-}\left(C^{r}\left(U_{i}\right)\right)\right)$	A function, defined for the for the stable component $S^{-}\left(C^{r}\left(U_{i}\right)\right)$, as $F\left(S^{-}\left(C^{r}\left(U_{i}\right)\right)\right)=\frac{\partial^{2}}{\partial A\partial B}\lambda {\left.\left(A,B,W\right)\right|}_{A=B=\bar{X}}>0$.

The notations of the adaptive dynamics model are included in Table A2.

**Table A2:** Summary of adaptive dynamics model notations.

Notation	Description
$\theta_{i}^{r}$	Gate parameter of unitary $U_{i}$ in an *r*-th measurement round.
$C^{r}\left(U_{i}\right)$	Objective function component associated with $U_{i}$ in an *r*-th measurement round
$f\left(C^{r}\left(U_{i}\right)\right)$	Objective function update function, $f\left(C^{r}\left(U_{i}\right)\right)=p_{i}^{r}C^{r-1}\left(U_{i}\right)+\left(1-p_{i}^{r}\right)C^{r}\left(U_{i}\right),$ where $C^{r-1}\left(U_{i}\right)$ is the objective function component at $U_{i}=I$, while $C^{r}\left(U_{i}\right)$ is an updated objective function component if $U_{i}\neq I$.
*A*	$A=C^{r-1}\left(U_{i}\right)={\bar{X}}_{i}^{r-1}+\rho_{i}^{r-1}\cos\left(\theta_{i}^{r}\right)$, where $\bar{X}$ and ρ are some constants.
*B*	$B=C^{r}\left(U_{i}\right)={\bar{X}}_{i}^{r}+\rho_{i}^{r}\sin\left(\theta_{i}^{r}\right)$, where $\bar{X}$ and ρ are some constants.
$C^{*}$	Target objective function value subject to be reached in *R* measurement rounds.
$C_{i}^{*}$	Target objective function component set for $U_{i}$.
$U({\vec{\theta }}^{r-1})$	A unitary sequence of an $\left(r-1\right)$-th measurement round, $U\left({\vec{\theta }}^{r-1}\right)=U_{L}^{r-1}\left(\theta_{L}^{r-1}\right)U_{L-1}^{r-1}\left(\theta_{L-1}^{r-1}\right)\ldots U_{1}^{r-1}\left(\theta_{1}^{r-1}\right)$.
$\alpha^{r-1}\left({\vec{\theta }}^{r-1}\right)$	Objective function component vector at $U({\vec{\theta }}^{r-1})$, $\alpha^{r-1}\left({\vec{\theta }}^{r-1}\right)={\left(C^{r-1}\left(U_{1}\right),\ldots ,C^{r-1}\left(U_{L}\right)\right)}^{T}$.
$\beta^{r}\left({\vec{\theta }}^{r}\right)$	Objective function component vector at $U({\vec{\theta }}^{r})$, $\beta^{r}\left({\vec{\theta }}^{r}\right)={\left(C^{r}\left(U_{1}\right),\ldots ,C^{r}\left(U_{L}\right)\right)}^{T}$.
$N_{QG}^{r-1}$	Vector of the total $n_{QG}$, $n_{QG}\geq L$ objective function components at an $\left(r-1\right)$-th measurement round, $N_{QG}^{r-1}={\left(C^{r-1}\left(U_{1}\right),\ldots ,C^{r-1}\left(U_{n_{QG}}\right)\right)}^{T}.$
$\kappa^{r-1}$	A vector in an $\left(r-1\right)$-th measurement round, defined as $\kappa^{r-1}=N_{QG}^{r-1}-\alpha^{r-1}\left({\vec{\theta }}^{r-1}\right)$.
$\tau^{r}$	An *r*-th measurement round, $\tau^{r}=N_{QG}^{r}-\beta^{r}\left({\vec{\theta }}^{r}\right)$.
$J\left(C^{r-1}\left(U_{i}\right),\tau^{r}\right)$	A Jacobian matrix at a particular $C^{r-1}\left(U_{i}\right)$ and $\tau^{r}$.
${\tilde{\alpha }}^{r-1}\left({\vec{\theta }}^{r-1}\right)$	A vector, identifies $\alpha^{r-1}\left({\vec{\theta }}^{r-1}\right)$ at an equilibrium state.
${\tilde{\kappa }}^{r-1}$	A vector referring to vector $\kappa^{r-1}$ at an equilibrium state.
$f\left(\cdot \right)$	A function evaluating the $\alpha^{r-1}{\left({\vec{\theta }}^{r-1}\right)}^{\prime }$ derivative of $\alpha^{r-1}\left({\vec{\theta }}^{r-1}\right)$.
$F\left(\cdot \right)$	A function evaluating the ${\kappa^{r-1}}^{\prime }$ derivative of $\kappa^{r-1}$.
$\lambda_{1}$	A coefficient identifying the number of non-zero $C^{r-1}\left(U_{1},\ldots ,U_{L}\right)$ objective function components in an $\left(r-1\right)$-th measurement round taken over unitaries $U_{1},\ldots ,U_{L}$.
$\lambda_{2}$	A coefficient referring to the number of zero $C^{r-1}\left(U_{1},\ldots ,U_{L}\right)$ objective function components of an $\left(r-1\right)$-th measurement round taken over $U_{1},\ldots ,U_{L}$.
S	State space, $S≐(\alpha^{r-1}\left({\vec{\theta }}^{r-1}\right),\beta^{r}\left({\vec{\theta }}^{r}\right),\kappa^{r-1})$.
$\tilde{S}$	Equilibrium state $\tilde{S}$, $\tilde{S}≐({\tilde{\alpha }}^{r-1}\left({\vec{\theta }}^{r-1}\right),0,{\tilde{\kappa }}^{r-1})$.
$J_{\tilde{S}}\left(\cdot \right)$	Jacobian matrix at the equilibrium $\tilde{S}$, $J_{\tilde{S}}\left(C^{r-1}\left(U_{i}\right),C^{r}\left(U_{i}\right),\tau^{r}\right)$.
$\lambda \left(\cdot \right)$	An eigenvalue of $J_{\tilde{S}}\left(C^{r-1}\left(U_{i}\right),C^{r}\left(U_{i}\right),\tau^{r}\right)$, $\lambda \left(C^{r-1}\left(U_{i}\right),C^{r}\left(U_{i}\right),\tau^{r}\right)$.
$S\left(C^{r}\left(U_{i}\right)\right)$	Stability of objective function component $C^{r}\left(U_{i}\right)$, $S\left(C^{r}\left(U_{i}\right)\right)=\mathrm{sign}\left(\lambda \left(C^{r-1}\left(U_{i}\right),C^{r}\left(U_{i}\right),\tau^{r}\right)\right)$.
$S^{-}\left(C^{r}\left(U_{i}\right)\right)$	A stable $S\left(C^{r}\left(U_{i}\right)\right)$ function.
$S^{+}\left(C^{r}\left(U_{i}\right)\right)$	An unstable $S\left(C^{r}\left(U_{i}\right)\right)$ function.
$f_{\varepsilon }\left(C^{r-1}\left(U_{i}\right)\right)$	Evolution function (canonical equation) of an objective function component $C^{r-1}\left(U_{i}\right)$.
$X\left(C^{r}\left(U_{i}\right)\right)$	Coefficient defined for $C^{r}\left(U_{i}\right)$, $X\left(C^{r}\left(U_{i}\right)\right)=\frac{\partial }{\partial C^{r}\left(U_{i}\right)}\lambda {\left.\left(C^{r-1}\left(U_{i}\right),C^{r}\left(U_{i}\right),\tau^{r}\right)\right|}_{A=B}.$
$\mu \left(C^{r-1}\left(U_{i}\right)\right)$	A ratio of probabilities.
$f_{b}\left(\cdot \right)$	A rate function.
$\sigma^{2}\left(C^{r-1}\left(U_{i}\right)\right)$	A term in the canonical equation.
ε	A constant.
$F\left(\cdot \right)$	A distribution family, $F\left(C^{r-1}\left(U_{i}\right),C^{r}\left(U_{i}\right)-C^{r-1}\left(U_{i}\right)\right)$.
$D\left(\cdot \right)$	A probability distribution with a standard deviation $\sigma =\sigma \left(C^{r-1}\left(U_{i}\right)\right)$, $D\left(C^{r-1}\left(U_{i}\right),C^{r}\left(U_{i}\right)-C^{r-1}\left(U_{i}\right)\right)$.
$\Delta_{\varepsilon }$	Distance between the objective function component $C^{r}\left(U_{i}\right)$ from a target value $C^{*}\left(U_{i}\right)$.
ζ	A constant.
$\varphi \left(C^{r-1}\left(U_{i}\right)\right)$	Probability that the objective function component is updated from $C^{r-1}\left(U_{i}\right)$ to $C^{r}\left(U_{i}\right)$ such that the value of $C^{r}\left(U_{i}\right)$ at an time interval $\left[t,t+dt\right]$ is in the interval of $\left[C^{r}\left(U_{i}\right),C^{r}\left(U_{i}\right)+dC^{r}\left(U_{i}\right)\right]$.
*W*	A vector referring to $\tau^{r}$ in an equilibrium state as $W={\tilde{\tau }}^{r}$.
$\Psi \left(C^{r-1}\left(U_{i}\right)\right)$	Coefficient for $C^{r-1}\left(U_{i}\right)$, $\Psi \left(C^{r-1}\left(U_{i}\right)\right)=\frac{\partial^{2}}{\partial A^{2}}\lambda {\left.\left(A,\bar{X},W\right)\right|}_{A=\bar{X}}$.
$\Psi \left(C^{r}\left(U_{i}\right)\right)$	Coefficient for $C^{r}\left(U_{i}\right)$, $\Psi \left(C^{r}\left(U_{i}\right)\right)=\frac{\partial^{2}}{\partial B^{2}}\lambda {\left.\left(\bar{X},B,W\right)\right|}_{B=\bar{X}}$.
$\tilde{X}\left(C^{r-1}\left(U_{i}\right)\right)$	Coefficient for $C^{r-1}\left(U_{i}\right)$ at an equilibrium, $\tilde{X}\left(C^{r-1}\left(U_{i}\right)\right)=\frac{\partial }{\partial A}\lambda {\left.\left(A,B,W\right)\right|}_{B=A}$.
$\tilde{X}\left(C^{r}\left(U_{i}\right)\right)$	Coefficient for $C^{r}\left(U_{i}\right)$ at an equilibrium, $\tilde{X}\left(C^{r}\left(U_{i}\right)\right)=\frac{\partial }{\partial B}\lambda {\left.\left(A,B,W\right)\right|}_{B=A}$.
$\zeta_{i}^{r}$	A gate parameter value defined for a unitary $U_{i}$ in an *r*-th measurement round.
$S^{\prime }\left(C^{r}\left(U_{i}\right)\right)$	Superposition of stability functions with respect to a particular objective function component $C^{r}\left(U_{i}\right)$, $S^{\prime }\left(C^{r}\left(U_{i}\right)\right)=pS^{+}\left(C^{r}\left(U_{i}\right)\right)+\left(1-p\right)S^{-}\left(C^{r}\left(U_{i}\right)\right)$, where *p* is the probability of an unstable stability function $S^{+}\left(C^{r}\left(U_{i}\right)\right)$.
$F\left(S^{+}\left(C^{r}\left(U_{i}\right)\right)\right)$	A function, defined for the for the $S^{+}\left(C^{r}\left(U_{i}\right)\right)$ unstable component, $F\left(S^{+}\left(C^{r}\left(U_{i}\right)\right)\right)=\Psi \left(C^{r-1}\left(U_{i}\right)\right)+\Psi \left(C^{r}\left(U_{i}\right)\right)>0$.
$F\left(S^{-}\left(C^{r}\left(U_{i}\right)\right)\right)$	A function, defined for the for the stable component $S^{-}\left(C^{r}\left(U_{i}\right)\right)$, as $F\left(S^{-}\left(C^{r}\left(U_{i}\right)\right)\right)=\frac{\partial^{2}}{\partial A\partial B}\lambda {\left.\left(A,B,W\right)\right|}_{A=B=\bar{X}}>0$.
